# Genes and Proteomes Associated With Increased Mutation Frequency and Multidrug Resistance of Naturally Occurring Mismatch Repair-Deficient *Salmonella* Hypermutators

**DOI:** 10.3389/fmicb.2020.00770

**Published:** 2020-05-08

**Authors:** Huanjing Sheng, Jinling Huang, Zhaoyu Han, Mi Liu, Zexun Lü, Qian Zhang, Jinlei Zhang, Jun Yang, Shenghui Cui, Baowei Yang

**Affiliations:** ^1^College of Food Science and Engineering, Northwest A&F University, Xianyang, China; ^2^School of Pharmaceutical Sciences, Jiangnan University, Wuxi, China; ^3^National Institutes for Food and Drug Control, Beijing, China

**Keywords:** mismatch repair, *Salmonella*, hypermutator, antibiotic resistance, proteomes

## Abstract

The emergence of antibiotic-resistant *Salmonella* through mutations led to mismatch repair (MMR) deficiency that represents a potential hazard to public health. Here, four representative MMR-deficient *Salmonella* hypermutator strains and *Salmonella* Typhimurium LT2 were used to comprehensively reveal the influence of MMR deficiency on antibiotic resistance among *Salmonella*. Our results indicated that the mutation frequency ranged from 3.39 × 10^–4^ to 5.46 × 10^–2^ in the hypermutator. Mutation sites in MutS, MutL, MutT, and UvrD of the four hypermutators were all located in the essential and core functional regions. Mutation frequency of the hypermutator was most highly correlated with the extent of mutation in MutS. Mutations in MMR genes (*mut*S, *mut*T, *mut*L, and *uvr*D) were correlated with increased mutation in antibiotic resistance genes, and the extent of antibiotic resistance was significantly correlated with the number of mutation sites in MutL and in ParC. The number of mutation sites in MMR genes and antibiotic resistance genes exhibited a significant positive correlation with the number of antibiotics resisted and with expression levels of *mut*S, *mut*T, and *mut*L. Compared to *Salmonella* Typhimurium LT2, a total of 137 differentially expressed and 110 specifically expressed proteins were identified in the four hypermutators. Functional enrichment analysis indicated that the proteins significantly overexpressed in the hypermutators primarily associated with translation and stress response. Interaction network analysis revealed that the ribosome pathway might be a critical factor for high mutation frequency and multidrug resistance in MMR-deficient *Salmonella* hypermutators. These results help elucidate the mutational dynamics that lead to hypermutation, antibiotic resistance, and activation of stress response pathways in *Salmonella*.

## Introduction

Bacteria carry several elaborate error prevention and correction systems to repair the accumulation of mutations that would be disadvantageous or deleterious (Lenhart et al., 2012; Li et al., 2018). However, spontaneous mutation always occurs naturally in bacterial populations, with a general frequency around 10^–10^ per replicated base pair (Chopra et al., 2003; Kaufmann and Hung, 2010; Morero et al., 2011; Chu et al., 2017). Some bacteria species have naturally occurring stable strong hypermutators, such as 1% of *Escherichia coli* and *Salmonella* strains, that can exhibit up to 1,000-fold increase in permanent mutation frequency over normal strains (LeClerc et al., 1996; Chopra et al., 2003; Yang et al., 2009; Henderson-Begg et al., 2010; Morero et al., 2011). In addition, 20% of *Pseudomonas aeruginosa* and 14% of *Staphylococcus aureus* isolates recovered from the lungs of patients with cystic fibrosis were found to be stable and heritable mutators with high mutation frequency and therefore can be classified as hypermutators (LeClerc et al., 1996; Matic et al., 1997; Oliver et al., 2000).

Although many genetic deficiencies can lead to a hypermutable phenotype (LeClerc et al., 1996; Eliopoulos and Blázquez, 2003), hypermutation of bacteria is primarily caused by deficiencies in *mut*S, *mut*H, *mut*L, and *mut*U (*uvr*D) within the mismatch repair (MMR) system (LeClerc et al., 1996; Jayaraman, 2009; Veschetti et al., 2020). Mutations in these genes can result in increased mutation frequency and enhanced interspecies recombination (Verma et al., 2009; Zhou et al., 2020). Consequently, some genes encoding for antibiotic sensitivity and/or resistance, antibiotic uptake and/or efflux systems, or their regulatory factors can be altered in MMR-deficient bacteria (Eliopoulos and Blázquez, 2003; Fàbrega et al., 2009; Li et al., 2012; Zhou et al., 2020).

*Salmonella* hypermutators with MMR deficiency have been reported among clinical and foodborne isolates, but this phenotype does not correlate strongly with antibiotic resistance (Henderson-Begg et al., 2010). For example, similar drug-resistant phenotypes were observed among *Salmonella* hypermutators with both low and high mutation frequencies (Yang et al., 2009). In addition, *Salmonella* hypermutators that lack defective MMR have been reported, despite insufficient numbers of isolates to determine whether the association was significant (Wang et al., 2015a). However, to what extent and through which central pathways mutations in the main MMR genes (*mut*S, *mut*L, *mut*H, and *uvr*D) influence mutation frequency and antibiotic susceptibility in *Salmonella* hypermutators has not been well investigated.

In this study, we analyzed the overall relationships among mutation frequency, antibiotic resistance, mutation and expression of functional genes, and signatures of differentially and specifically expressed proteins in MMR-deficient *Salmonella* hypermutators. The objective of this study was to reveal how MMR deficiency influences hypermutation and antibiotic resistance in *Salmonella*.

## Materials and Methods

### *Salmonella* Strains

Four representative strains used in this study were screened from 96 *Salmonella* hypermutators and selected according to their mutation frequencies, amino acid substitutions in MutS, MutL, MutT, and UvrD within the MMR system, pulsed-field gel electrophoresis (PFGE) profiles, antibiotic resistance phenotypes, food sources, and sampling regions. The 96 hypermutators were previously screened from 1,264 *Salmonella* isolates, including 1,151 from retail chicken, 64 from pork, 13 from cold dish, and 12 from clinical salmonellosis, nine from mutton, seven from beef, five from fish, two from steamed stuffed bun, and one from dumpling. The serotype, antibiotic susceptibility, and PFGE profile of each isolate were described in our earlier studies (Wang et al., 2015a, b; Yang et al., 2010, 2013). In addition, the non-hypermutator *Salmonella* Typhimurium LT2 was used as a control strain for gene amplification, sequencing, and expression analyses and for all protein analyses.

### Hypermutator Screening

*Salmonella* hypermutators were screened as previously described (LeClerc et al., 1996). Briefly, single colonies of each *Salmonella* isolate grown on Luria–Bertani (LB) agar (BD, Cockeysville, MD, United States) were collected and cultured in 5 ml of LB broth (BD) at 37°C, with shaking at 100 rpm for 18–24 h. Then, 100 μl of 10^3^-, 10^4^-, 10^5^-, and 10^6^-fold diluted broth culture was spread in triplicate on LB agar (BD) plates containing 100 μg/ml rifampicin (Sigma, St. Louis, MO, United States) and 20 μg/ml nalidixic acid (Sigma), respectively. Meanwhile, 100 μl of 10^6^-, 10^7^-, and 10^8^-fold diluted broth culture was spread in triplicate on antibiotic-free LB agar (BD) plates. After incubation (37°C, 18–24 h), the isolates yielding more than 50 separated colonies on each antibiotic-containing plate were selected as hypermutators. The mutation frequency was estimated by dividing the number of colony-forming units (CFUs) on antibiotic-containing plates by the number of CFU on antibiotic-free plates. All putative hypermutators were retested at least twice (LeClerc et al., 1996; Yang et al., 2009). *Salmonella* SL226 was used as a positive control for hypermutator screening (Yang et al., 2009).

### Mismatch Repair Deficiency Analysis

The *mut*S gene was amplified from each *Salmonella* hypermutator strain using a long-range PCR kit (LA PCR^TM^ Genome DNA kit; TaKaRa, Dalian, China) with the following primers (these primers were also used for *mut*S sequencing): *mut*S-F (5′-GACTGAGTGCAGGCTTAACAT TGATACTTA-3′) and *mut*S-R (5′-CCATGCGGCGATTGAG AACTGGCTTAGTAA3′). Primers used for *mut*L, *mut*H, and *uvr*D amplification and sequencing are listed in Supplementary Table S1. DNA sequences were translated into amino acid sequences using Primer Premier 5 (Premier Biosoft International, Palo Alto, CA, United States). Mutations in MutS, MutL, MutH, and UvrD were detected by comparing their amino acid sequences with those in *Salmonella* Typhimurium LT2. Hypermutator strains with any amino acid mutation or deletion in their MutS, MutL, MutH, and UvrD sequences were considered to be MMR deficient.

To determine whether the conserved domains of MutS, MutL, MutH, and UvrD are affected by amino acid mutation, the deduced protein structures of mutated MutS, MutL, MutT, and UvrD were analyzed online using a conserved domain database^1^ with default parameters. The corresponding amino acid sequences of these proteins from *Salmonella* Typhimurium LT2 (GCA_000006945.2) were used as control sequences.

### Mutation Analysis of Genes Associated With Antibiotic Resistance and DNA Replication Repair

Template DNA was extracted from *Salmonella* cultures, as previously described (Sambrook et al., 1989). Briefly, the four hypermutator strains and *Salmonella* Typhimurium LT2 were cultured on LB agar (BD) plates at 37°C for 18–24 h. Then, one or two colonies from each strain were randomly selected and suspended in 1 ml of sterilized distilled water, heated to 100°C for 10 min, and centrifuged at 15,000 × *g* for 10 min. The supernatant was carefully collected and used as DNA template for PCR amplification of functional genes associated with antibiotic resistance and DNA replication repair.

Primers (Supplementary Table S1) were synthesized by AuGCT DNA-SYN Biotechnology Co., Ltd. (Beijing, China). PCR amplification was conducted in 50-μl reaction mixtures containing 0.6 μM of each primer, 2.5 U of Premix Taq^TM^ DNA polymerase (ExTaq^TM^ Version 2.0; TaKaRa), and 500 ng of template DNA on a MyCycle PCR system (Bio-Rad, Hercules, CA, United States). The PCR program was set as follows: an initial denaturation step at 94°C for 10 min; 35 cycles of 94°C denaturation for 1 min, annealing temperature for 1 min, and extension at 72°C for 1 min; and a final extension of 72°C for 10 min. PCR products were sequenced by Sunny Biotechnology Co., Ltd. (Shanghai, China). DNA sequences were aligned and analyzed using the NCBI BLAST program^2^.

### Expression Analysis of Genes Associated With Antibiotic Resistance and DNA Replication Repair

The expression levels of four genes in the MMR system and 15 genes associated with antibiotic resistance were determined by real-time quantitative PCR (RT-qPCR). Total RNA was extracted from cultures of the four hypermutator strains and *Salmonella* Typhimurium LT2 using an RNAprep Pure Cell/Bacteria Kit (TIANGEN, Beijing, China), according to the manufacturer’s instructions. Briefly, 1.5 ml of *Salmonella* LB (BD) cultures with an optical density at 600 nm of 0.5 were centrifuged at 13,000 × *g* for 2 min at 4°C. Cells were then resuspended in 1 ml of Tris-EDTA buffer with lysostaphin (1 mg/ml; Sigma) and incubated at 37°C for 10 min. The lysate was transferred into a column for total RNA extraction.

The extracted RNA was treated with RNase-free DNase I at 37°C for 10 min. The quality and concentration of the RNA were determined using a nucleic acid and protein mini-spectrophotometer (Nano-200; Allsheng Instruments Co., Ltd., Hangzhou, China). RNA was reverse transcribed to generate cDNA using a PrimeScript RT reagent Kit (Perfect Real Time; TaKaRa), according to the manufacturer’s instructions. The cDNA was stored at −20°C until use.

Primers used for RT-qPCR are listed in Supplementary Table S1. RT-qPCR was carried out in an iQ5 Real-time PCR Detection System (Bio-Rad) in 25 μl reaction mixtures containing 1 × SYBR^®^ *Premix Ex Taq^TM^* II, 0.4 μM of each primer, 100 ng of template DNA, and 8.5 μl of double-distilled water. The following PCR program was used: initial denaturation at 95°C for 30 s; 40 cycles of 95°C for 5 s and 60°C for 60 s; and a dissociation step, with 71 cycles of 95°C for 15 s and 60°C for 30 s. The expression level of each gene was determined in triplicate and normalized to expression of gyrase subunit B (*gyr*B) by the 2^–ΔΔ*CT*^ method, as described previously (Li et al., 2014). Genes with greater than twofold change in their relative expression levels were considered to be upregulated or downregulated.

### Protein Extraction

Sample preparation and protein extraction were carried out according to previously described methods (Han et al., 2013), with some modifications. The four hypermutator strains and *Salmonella* Typhimurium LT2 were each streaked on LB agar (BD) plates and incubated at 37°C. Afterward, a single colony from each strain was subcultured in 5 ml of LB broth (BD) at 37°C for 14 h. Then, 0.05 ml of the subcultures were added into 10 ml of LB broth (BD) and incubated at 37°C for another 5 h with shaking at 120 rpm. Cells were harvested by centrifugation at 4°C for 5 min at 15,000 × *g*, and the pellets were washed three times using 4°C distilled water.

The cells were then resuspended in lysis buffer containing 7 M urea, 2% (w/v) chaps, 2% (v/v, pH 3–10) pharmalyte, 1% (w/v) dithiothreitol, and 5 mM pefabloc (proteinase inhibitor). Subsequently, the cell suspensions were incubated on ice for 15 min and sonicated until clear solutions were obtained. After centrifugation (4°C, 15,000 × *g*, 15 min), the supernatants were collected. Protein concentrations in the supernatants were measured using a Bradford Protein Assay Kit (TaKaRa).

### Protein Separation *via* 2-Dimensional Electrophoresis and Image Analysis

Two-dimensional electrophoresis (2-DE) was used to identify the differentially and specifically expressed proteins in the MMR-deficient *Salmonella* hypermutators. Proteins from the four hypermutator strains and *Salmonella* Typhimurium LT2 were separated using 2-DE (Bio-Rad), according to the procedures of the Bio-Rad Proteome Works^TM^ System (Bio-Rad Proteome Works^TM^ System-Proteomics 2007), with some modifications. Briefly, ∼1.2 mg of total protein was dissolved in a rehydration buffer (lysis buffer with 0.001% bromophenol blue). Then, 400 μl of the protein solution was loaded onto an immobilized pH gradient strip (17 cm, pH 3–10, linear; Bio-Rad). The strip was subsequently rehydrated for 14 h in a protean isoelectric focusing cell (Bio-Rad). Rehydration was carried out as follows: 250 V for 30 min, 1,000 V for 1 h, 10,000 V for 5 h, 10,000 V for 60,000 V⋅h.

Before separation in the second dimension, the IPG strip was equilibrated in buffer I [6 M urea, 1% (w/v) sodium dodecyl sulfate (SDS), 30% (v/v) glycerol, 50 mM Tris-Cl (pH 8.8), and 1% (w/v) dithiothreitol] for 15 min, then equilibrated in buffer II [6 M urea, 1% (w/v) SDS, 30% (v/v) glycerol, 50 mM Tris-Cl (pH 8.8), and 2.5 mM iodoacetamide] for 15 min. After equilibration, the strip was immediately transferred into a 12.5% SDS–polyacrylamide gel electrophoresis (SDS–PAGE) gel using an Ettan DALT six Large Vertical System (GE Healthcare, United States). Initially, SDS-PAGE was run at 1 W/gel for 1 h and then increased to 13 W/gel until the bromophenol blue dye (Sigma) migrated out of the bottom of the gel. For each *Salmonella* strain, 2-DE was performed on three biological replicates. After separation, the gel was stained using Coomassie blue brilliant (R-250), destained, and scanned using an image scanner (Bio-Rad). The resulting images were analyzed using PDQuest (version 8.0, Bio-Rad).

### Trypsin Digestion and Protein Identification by Mass Spectrometry

To comprehensively investigate the contribution of differentially and/or specifically expressed proteins to changes in gene expression, hypermutation, and antibiotic resistance, the differentially and/or specifically expressed proteins acquired from 2-DE were further identified using high-performance liquid chromatography–chip/electrospray ionization–quadrupole time-of-flight/tandem mass spectrometry (HPLC-Chip/ESI-Q-TOF/MS/MS). Protein digestion and peptide extraction from 2-DE gel were performed according to a previously established protocol (Han et al., 2013). Briefly, the differentially and specifically expressed proteins from the hypermutator strains were washed in 100 ml of destaining solution containing 50% acetonitrile and 25 mM NH_4_HCO_3_ until the gel was transparent. The colorless gel was then dehydrated for 10 min and dried for 30 min using a Speed-Vac system (RVC 2–18; Marin Christ, Germany). Subsequently, 10 μl of trypsin solution (final concentration = 10 ng/μl) was added to each dried gel spot to digest the proteins. The digested proteins were analyzed using an HPLC-Chip/ESI-Q-TOF/MS/MS system (Q-TOF 6520; Agilent Technologies, Santa Clara, CA, United States) equipped with 1200 series nanoflow and HPLC-Chip interface (Chip-cube G4240A; Agilent Technologies).

The peptides were separated by reversed phase chromatography using a microfluidic chip, which consisted of a 160-nl trap column (5 mm) and an analytical column (75 μm i.d., 150 mm length, with a 300 A C18 stationary phase). All data were acquired in the positive ionization mode with the mass to charge ratio (m/z) between 300 and 2,000. MS was operated in auto MS/MS acquisition mode, and the top three most intense precursor ions were selected. The peptide was loaded in 0.1% formic acid at 4 μl/min and resolved at 500 nl/min for 15 min. Elution from the analytical column was performed by binary solvent mixture composed of water with 0.1% formic acid (solvent A) and acetonitrile with 0.1% formic acid (solvent B). The following gradients were used: from 3 to 8% of solvent B for 1 min, from 8 to 40% of solvent B for 5 min, from 40 to 85% of solvent B for 1 min, and 85% of solvent B for 1 min.

MS/MS spectra were retrieved using Peaks 7.0 (Bioinformatics Solutions Inc., Waterloo, ON, Canada) and searched in-house using Mascot Distiller (version 2.3; Matrix Science, United Kingdom) and a common repository of adventitious proteins (cRAP, from The Global Proteome Machine Organization, downloaded in May 2011) with 315,327 entries in total. The search parameter of carbamidomethyl (C) was selected as fixed modification (57.02), whereas oxidation (M) was selected as variable modification (15.99). The following search parameters were used in other sets: taxonomy, all entries; enzyme, trypsin; missed cleavage, 1; precursor ion mass tolerance, ±50 ppm; and fragment ion mass tolerance, ±0.05 Da. The results of protein identification were accepted if they contained at least two identified peptides having both a minimal cutoff Mascot score of 24 and a 95% probability of correct matching.

### Data Analysis

The definition and category of the differentially and specifically expressed proteins were ascertained using the Uniprot database^3^ and BioCyc database^4^. To further annotate the biological roles of the 19 functional genes and the differentially or specifically expressed proteins, multiple functional analyses were performed using the Database for Annotation, Visualization and Integrated Discovery (DAVID, version 6.8) tool^5^ (Sherman and Lempicki, 2009). Gene Ontology (GO) terms that were selected included “biological process,” “cell component,” and “molecular function” annotations. The Kyoto Encyclopedia of Genes and Genomes (KEGG) database and the KEGG Automatic Annotation Server (KAAS) program^6^ were used to enrich the possible metabolic pathways that these genes and proteins are involved in. GO terms and KEGG pathways with *P-*values < 0.05 were considered significant. The GO and KEGG results were visually represented in a circos plot generated using the R package GOplot (version 1.0.2). For interaction network analysis, all detected genes and proteins in the hypermutator strains were analyzed using the STRING database (version 10.5^7^), and the minimally required interaction score of the genes and proteins was set at medium confidence (0.400). The resulting network was visualized in Cytoscape (version 3.6.1^8^).

All measurement data are reported as means ± standard deviation of triplicate tests. Statistical analyses were performed using SPSS 19.0 (IBM Corp., New York, NY, United States). Differences in the expression levels of functional genes were assessed using one-sample *t*-tests. Spearman’s rank correlation analysis with one-sided *t*-test was used to examine potential relationships between mutation frequency, antibiotic resistance, amino acid substitutions, and expression of functional genes. A correlation matrix between mutation frequency, total number of mutations observed in genes, and the extent of antibiotic resistance (or the number of antibiotics resisted) was built in R using the corrplot package (version 0.84). In addition, a correlation matrix among mutation frequency, the number of mutation sites and number of antibiotics resisted, and the expression of functional genes was computed using the R package Hmisc (version 4.2-0). Clustering and correlations were visualized using the R package pheatmap (version 1.0.10^9^). *P-*values < 0.05 were considered statistically significant for all tests.

## Results

### *Salmonella* Hypermutators

**FIGURE 1:**
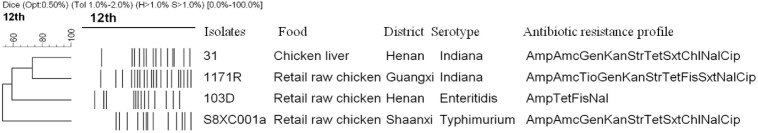
Dendrogram showing the pulsed-field gel electrophoresis profiles, food sources, sampling regions, serotypes, and antibiotic resistance genotypes of the four *Salmonella* hypermutators. Amp, ampicillin; Amc, amoxicillin–clavulanic acid; Gen, gentamicin; Kan, kanamycin; Amk, amikacin; Str, streptomycin; Fis, sulfisoxazole; Tio, ceftiofur; Tet, tetracycline; Sxt, sulfamethoxazole–trimethoprim; Chl, chloramphenicol; Nal, nalidixic acid; Cip, ciprofloxacin.

Ninety-six of the 1,264 *Salmonella* isolates obtained from retail foods were identified as MMR-deficient hypermutators. An amino acid substitution of Val246Ala in the MutS protein was detected in 56 of the 96 hypermutators, and the Val421Phe mutation in MutS was detected in one hypermutator. Four hypermutators (designated 31, 1171R, S8XC001a, and 103D) were selected as representative strains for subsequent analyses (Figure 1).

**FIGURE 2:**
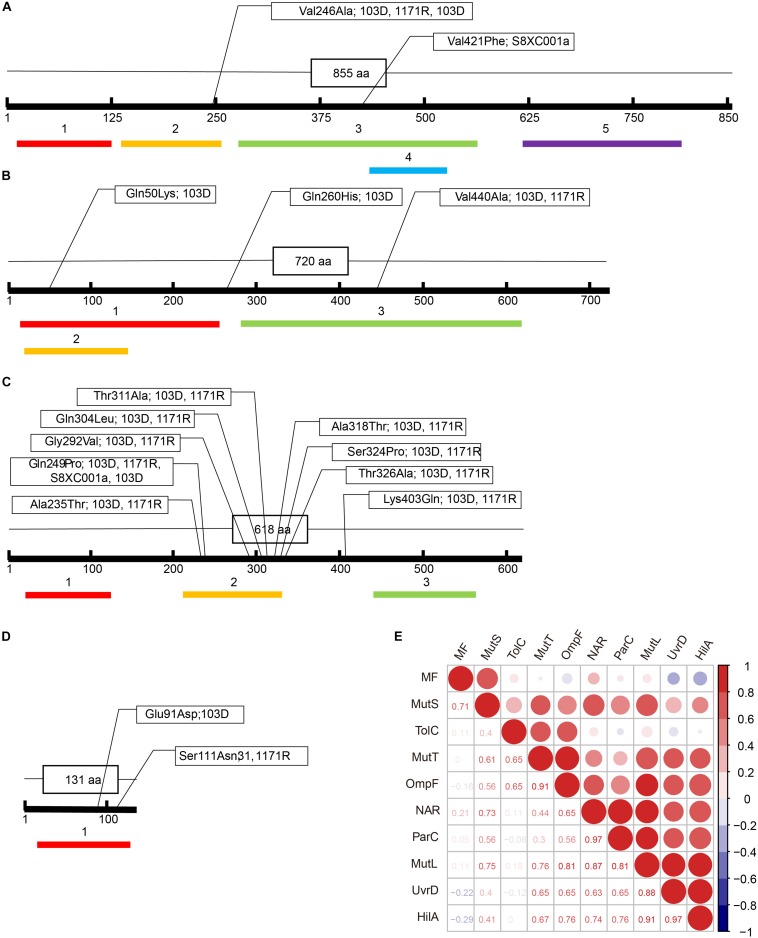
Amino acid (aa) substitutions detected in different domains of the MutS, MutL, MutT, and UvrD proteins in the four *Salmonella* hypermutators. **(A)** MutS. Colored boxes: 1: N-terminal domain (from aa 13 to 125); 2: Connector domain (from aa 133 to 258); 3: Core domain (from aa 274–561); 4: Clamp domain of core domain (from aa 430 to 521); and 5: C-terminal domain (from aa 612 to 799). **(B)** UvrD. Colored boxes: 1: N-terminal domain (from aa 10 to 272); 2: AAA domain (from aa 14 to 139), which contains a P-loop motif characterizing the AAA superfamily, with many members being conjugative transfer proteins; and 3: C-terminal domain (from aa 277 to 617). **(C)** MutL. Colored boxes: 1: Histidine kinase-like ATPase C-terminal domain (from aa 23 to 113); 2: Transducer domain, having a ribosomal S5 domain 2-like fold (from aa 210 to 330); and 3: C Terminal dimerization domain (from aa 442 to 565). **(D)** MutT. Colored boxes: 1: NUDIX domain (from aa 7 to 125). **(E)** Correlation matrix between amino acid substitutions, antibiotic resistance phenotype, and mutation frequency in the hypermutators. MF, mutation frequency; NAR, number of antibiotic resisted.

The Val246Ala amino acid mutation in MutS was detected in strains 31, 1171R, and 103D (located in the connector domain; Figure 2A), while the Val421Phe mutation was detected in strain S8XC001a (located in the core domain; Figure 2A). Mutation frequencies, as determined on LB–rifampicin (100 μg/ml) plates, for the different hypermutator strains were as follows: 1.08 × 10^–3^ for strain 31, 3.39 × 10^–4^ for strain 1171R, 5.46 × 10^–2^ for strain S8XC001a, and 1.32 × 10^–3^ for strain 103D (Supplementary Table S2).

### Mismatch Repair and Functional Gene Deficiency Analysis

Compared to the amino acid sequences of the MutS, MutL, MutT, UvrD, TolC, ParC, HilA, and OmpF proteins in *Salmonella* Typhimurium LT2, mutations were detected in these proteins in the hypermutator strains 31, 1171R, S8XC001a, and 103D (Supplementary Table S2).

Nine amino acid substitutions were identified in MutL, including eight located in the transducer domain, which has a ribosomal S5 domain 2-like fold (Ala235Thr, Gln249Pro, Gly292Val, Gln304Leu, Thr311Ala, Ala318Thr, Ser324Pro, and Thr326Ala), and an additional mutation (Lys403Gln) (Figure 2C). In the UvrD protein of strain 31, a Gln50Lys mutation was detected in the AAA domain, which contains a P-loop motif related to conjugative transfer, along with a Gln260His mutation in the N-terminal domain, and the Val440Ala mutation in the C-terminal domain (Figure 2B). In addition, a Glu91Asp mutation (strain 103D) and a Ser111Asn mutation (strain 31 and 1171R) were detected in the NUDIX domain of MutT (Figure 2D). Mutation sites in MutS, MutL, MutT, and UvrD of the four hypermutator strains were all located in the essential and core functional regions of the proteins (Figures 2A–D).

The extent of antibiotic resistance (i.e., the number of antibiotics resisted) was significantly correlated with the number of mutation sites in MutL (Spearman’s rank correlation, *R* = 0.856; one-sided *t*-test: *n* = 5, *P* = 0.03) and in ParC (*R* = 0.973; one-sided *t*-test: *n* = 5, *P* = 0.003). Similar correlations were observed for mutations in MutS (*R* = 0.725, *P* = 0.083), UvrD (*R* = 0.631, *P* = 0.127), OmpF (*R* = 0.649, *P* = 0.118), and HilA (*R* = 0.740, *P* = 0.076), albeit not statistically significant. However, although mutation frequency did not significantly correlate with the number of mutation sites observed in most genes, mutation frequency was most highly correlated with the extent of mutation in MutS (*R* = 0.707, *P* = 0.091; Figure 2E and Supplementary Table S3). Mutations in the MMR genes (*mut*S, *mut*T, *mut*L, and *uvr*D) were also correlated with increased mutation in antibiotic resistance genes (*par*C and *omp*F). However, no significant correlations were found between mutations in MutS, MutT, and UvrD and mutations in other MMR and antibiotic resistance genes.

### Functional Gene Expression

**FIGURE 3:**
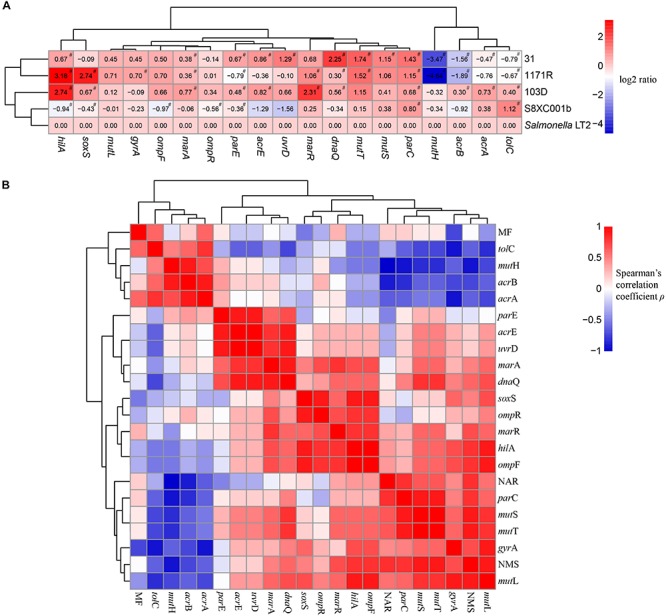
Clustering and correlation analysis revealing the influence of mismatch repair deficiency on mutation frequency and antibiotic susceptibility in the four *Salmonella* hypermutators. **(A)** Heatmap of log2 fold changes in the expression levels of 19 genes. Red color indicates upregulation, whereas blue color indicates downregulation. All pound signs (#) indicate statistical differences by one-sample *t*-test at *P* < 0.05 versus *Salmonella* Typhimurium LT2. **(B)** Clustering and correlation analysis visualizing the relationships among mutation frequency, the number of mutation sites and number of antibiotics resisted, and expression levels of 19 genes. Red and blue colors indicate positive and negative correlations, respectively. MF, mutation frequency; NAR, number of antibiotic resisted; NMS, number of mutation sites.

Clustering and correlation analysis revealed the influence of expression of mutated MMR genes on mutation frequency and antibiotic susceptibility in the *Salmonella* hypermutator strains. In strains 31 and 1171R, *mut*S, *mut*T, and *par*C were significantly upregulated (log2 ratio > 1, *P* < 0.05), whereas *mut*H and *acr*B were significantly downregulated (log2 ratio < −1, *P* < 0.05) compared to expression in *Salmonella* Typhimurium LT2 (Figure 3A).

The number of mutation sites exhibited a significant positive correlation with the number of antibiotics resisted (*R* = 0.821, *P* = 0.044) and with expression levels of *mut*S (*R* = 0.900, *P* = 0.019), *mutT* (*R* = 0. 009, *P* = 0.019), and *mut*L (*R* = 0.900, *P* = 0.019). However, *mut*H expression was negatively correlated with the number of mutation sites (*R* = −0.900, *P* = 0.019), the number of antibiotics resisted (*R* = −0.975, *P* = 0.002), and expression levels of *acr*B (*R* = −0.900, *P* = 0.019) and *par*C (*R* = −0.900, *P* = 0.019). In addition, *mut*H expression exhibited a relatively high correlation with expression levels of *mut*S (*R* = −0.800), *mut*T (*R* = −0.800), *mut*L (*R* = −0.700), and *acr*A (*R* = −0.700), although these correlations were not statistically significant (*P*-values = 0.052, 0.052, 0.094, and 0.094, respectively). Likewise, the expression levels of MMR genes (*mut*S, *mut*L, *mut*T, and *uvr*D) were closely related to those of most antibiotic resistance genes (*acr*E, *par*C, *par*E, *mar*A, and *dna*Q). Mutation frequency did not correlate with the expression levels of most genes (Figure 3B and Supplementary Table S4).

### Two-Dimensional Electrophoresis Profiles of Hypermutator Proteomes

**FIGURE 4:**
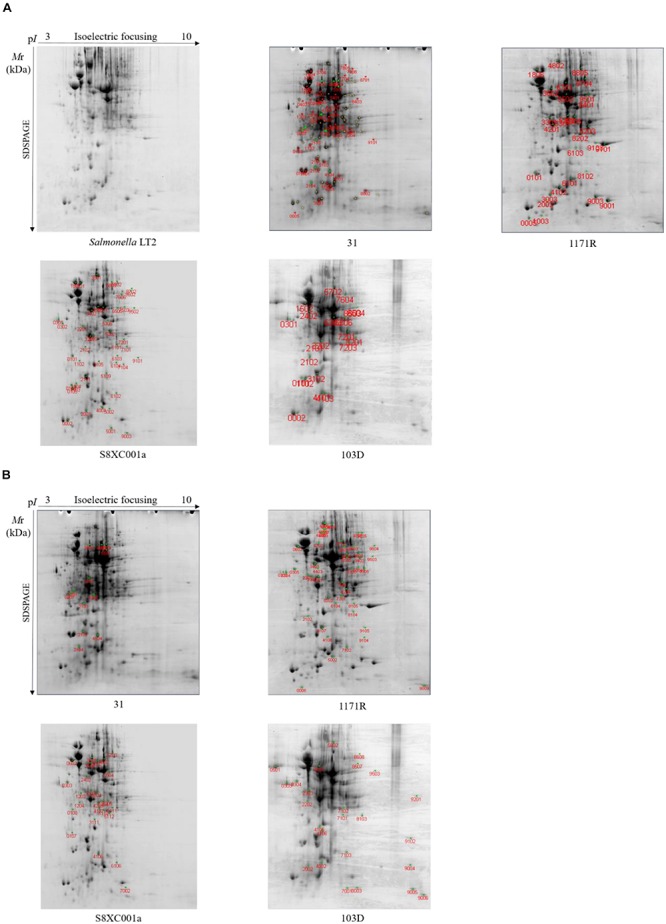
Two-dimensional electrophoresis profiles of the four *Salmonella* hypermutators. *Salmonella* Typhimurium LT2 served as a non-hypermutator control. **(A)** Differentially expressed protein spots with red labels. **(B)** Specifically expressed protein spots with red labels.

Compared to *Salmonella* Typhimurium LT2, a total of 42 differential protein spots were detected by 2-DE in hypermutator strain 31. Among these, the expression levels of 21 proteins were significantly upregulated (fold change > 2; *P* < 0.05), and expression levels of 21 proteins were significantly downregulated (fold change > 2; *P* < 0.05). In addition, 12 specific protein spots were detected in strain 31 (Figure 4).

Twenty-nine differentially expressed protein spots were identified in hypermutator strain 1171R, 22 of which were significantly upregulated (fold change > 2; *P* < 0.05) and seven were significantly downregulated (fold change > 2; *P* < 0.05). Moreover, 47 specific protein spots were detected in strain 1171R (Figure 4).

In hypermutator strain S8XC001a, 26 specific protein spots were detected, and 45 protein spots were found to be differentially expressed compared to *Salmonella* Typhimurium LT2. Among these proteins, 29 were upregulated (fold change > 2; *P* < 0.05) and 16 (fold change > 2; *P* < 0.05) were downregulated in strain S8XC001a (Figure 4).

In hypermutator strain 103D, there were 10 upregulated proteins (fold change > 2; *P* < 0.05), 11 downregulated proteins (fold change > 2; *P* < 0.05), and 25 specifically expressed proteins detected by 2-DE (Figure 4).

### Mass Spectrometry Identification of Non-redundant Proteins

**FIGURE 5:**
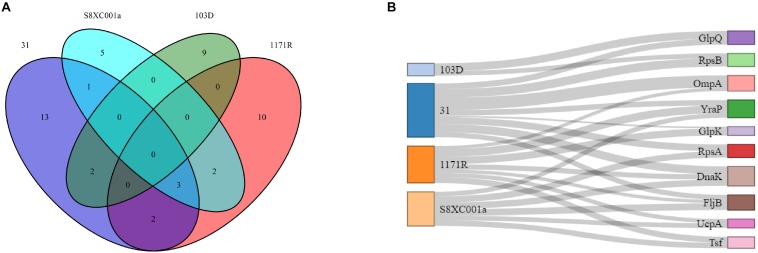
Comparison of non-redundant proteins identified in the four *Salmonella* hypermutators by mass spectrometry. **(A)** A Venn diagram showing the distribution of 47 non-redundant proteins. **(B)** A Sankey diagram showing the distribution of 10 common proteins, in which the thickness of the line indicates protein expression level.

In total, 47 non-redundant, differentially expressed proteins were identified by MS in the four hypermutator strains. Among these, 13, 10, 9, and 5 proteins were exclusively identified in strains 31, 1171R, 103D, and SBXC001a, respectively, and 10 proteins overlapped in the four strains (Figure 5A). Eight of the 10 common proteins (RpsB, OmpA, YraP, GlpK, RpsA, DnaK, UcpA, and Tsf) were upregulated (Figure 5B), except for FljB in strain 31 and GlpQ in strain 103D (Supplementary Table S5). The upregulated proteins included members of ribosomal protein family (RpsB, RpsA, RplL, RplI, RplO, and RpsF), glycerol kinase, SSU ribosomal protein S2p, molecular chaperone Dnak, short-chain dehydrogenase, periplasmic protein, and phosphoglycerate kinase. The downregulated proteins included elongation factor Ts, F0F1 ATP synthase subunit beta, 50S ribosomal protein L7/L12, lysyl-tRNA synthetase, alpha-helical coiled coil protein, and flagellin.

Proteins that were specifically expressed in strain 31 were identified to be elongation factor Tu, transcriptional regulator, OmpA glycerol kinase, and peptidase PmbA. Those in strain 1171R were identified to be OmpA, flagellin, periplasmic protein, and phosphoglyceromutase. In strain S8XC001a, the specifically expressed proteins included flagellar synthetase, isocitrate dehydrogenase, and YciF. In addition, transcription termination factor rho and cold shock protein were specifically expressed in strain 103D (Supplementary Table S5).

### Gene Ontology and Kyoto Encyclopedia of Genes and Genomes Functional Enrichment of Differentially/Specifically Expressed Proteins

**FIGURE 6:**
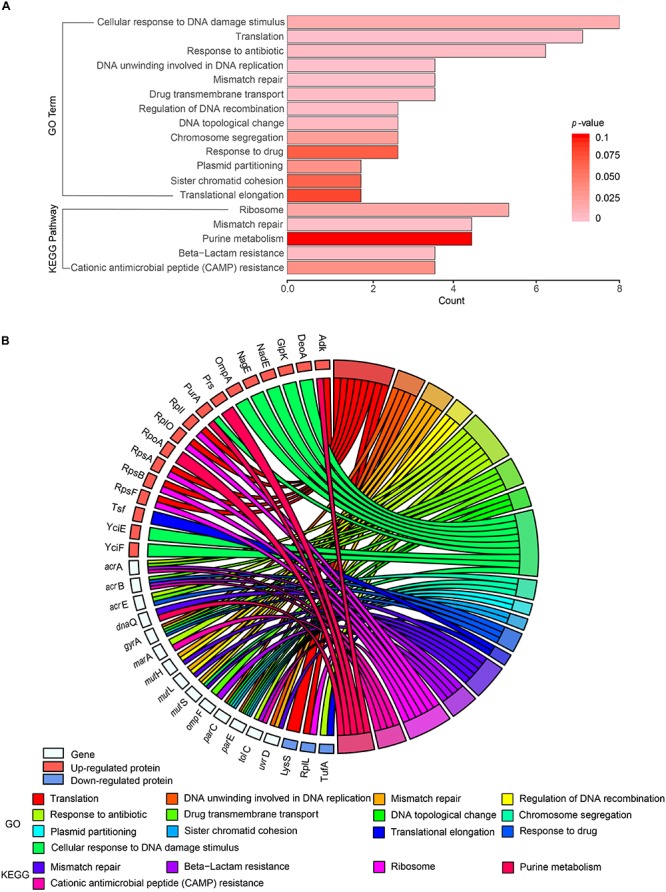
Gene Ontology (GO) and Kyoto Encyclopedia of Genes and Genomes (KEGG) functional enrichment analyses of 19 functional genes, differentially and specifically expressed proteins from the four *Salmonella* hypermutators. **(A)** Functional enrichment of proteins. Number of GO terms and their *P*-values are indicated by the *x*-axis and bar color, respectively. **(B)** Circos plot showing the relationship between proteins and GO terms. Protein expression is indicated by colored squares.

Cross-examination of GO enrichment analysis results indicated that a substantial number of the differentially/specifically expressed proteins and proteins encoded by the functional genes tested in this study were enriched in the same biological functions (Figure 6A). For example, TufA was found in the same terms as TolC, MarA, GyrA, AcrA, ParE, and AcrE (response to antibiotics), whereas GlpK, NadE, YciE, YciF, NagE, DeoA, and PurA appeared in the same terms as MutS and OmpA (cellular response to DNA damage stimulus). The proteins identified by MS were mainly related to cellular response to DNA damage stimulus, translation, and response to antibiotics (Figure 6A). Results of the biological process analysis also indicated that the identified proteins were significantly enriched for translation processes (RpsB, RpsA, RplL, LysS, Adk, RplI, RplO, and RpsF; Figures 6A,B).

KEGG pathway analysis showed that the ribosome pathway was significantly enriched in the differentially expressed proteins in the hypermutator strains. Six proteins (RpsB, RpsA, RplL, RplI, RplO, and RpsF) distributed among the large and small subunits of ribosome were involved in ribosome function (Supplementary Figure S1). Increased expression and significant upregulation (fold change > 2; *P* < 0.05) of ribosomal proteins were also found in the hypermutator strains (Supplementary Table S5). Moreover, some proteins identified in the hypermutator strains by MS were related to stress response to environmental changes, including oxidative stress (Lpd, Icd, AhpC, and SodA), heat (DnaK and SodA), chemical stimulus (SodA, AhpC, and DnaK), and response to organic substances (NagE and AhpC).

### Interaction Network Analysis

**FIGURE 7:**
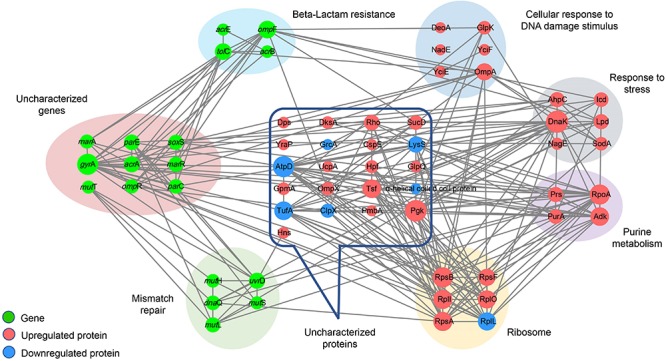
Interaction network of 19 functional genes and differentially/specifically expressed proteins in the four *Salmonella* hypermutators. The network is displayed using the STRING database (version 10.5; http://string-db.org/) and Cytoscape (version 3.6.1; https://cytoscape.org/) according to the “Grid” layout. Node size is proportional to the degree of the node. Gray lines indicate the interaction between genes and proteins.

Interaction network analysis indicated that, compared to *Salmonella* Typhimurium LT2, no significant changes in the expression of proteins encoded by the 19 functional genes were detected in the four hypermutator strains by MS. However, other proteins, including the 30S ribosomal protein S1, S2, and S6 families, glycerol kinase, molecular chaperone Dnak, short-chain dehydrogenase, periplasmic protein, phosphoglycerate kinase, elongation factor Ts, 50S ribosomal protein L7/L12, lysyl-tRNA synthetase, alpha-helical coiled coil protein, and flagellin, were commonly detected in the hypermutator strains. The MMR genes (*mut*H, *mut*L, *mut*S, and *uvr*D) not only exhibited internal interactions but also had direct relationships with antibiotic resistance genes (*par*C, *par*E, *gyr*A, and *dna*Q) as well as some differentially expressed proteins, including chaperone protein DnaK, DNA-binding transcriptional dual regulator H-NS, and adenylosuccinate synthetase PurA (Figure 7).

Interestingly, ribosome-related proteins formed core connections with other genes and proteins, including genes associated with MMR and antibiotic resistance, as well as proteins associated with purine metabolism, cellular response to DNA damage stimulus, and alpha-helical coiled coil. Therefore, in addition to the MMR system, ribosome and differentially/specifically expressed proteins associated with stress response and DNA damage stimulus appeared to be critical factors involved in high mutation frequency and multidrug resistance in MMR-deficient *Salmonella* hypermutators (Figure 7).

## Discussion

Mutation is an unavoidable consequence of errors produced during DNA replication (Eliopoulos and Blázquez, 2003; Jayaraman, 2009; Chu et al., 2017). According to previous studies (LeClerc et al., 1996; Matic et al., 1997; Oliver et al., 2000; Eliopoulos and Blázquez, 2003; Jayaraman, 2009; Chu et al., 2017), mutations in the MutS, MutL, MutH, and UvrD proteins of the MMR system are the most important factors leading to hypermutability in bacteria. The mutation frequency in these strains ranged from 3.39 × 10^–4^ to 5.46 × 10^–2^, which was increased by approximately 10^5^- to 10^6^-fold over the normal spontaneous mutation frequency of bacteria (LeClerc et al., 1996; Broaders et al., 2013; Zhou et al., 2020). In addition to high mutation frequency, MMR deficiency is associated with antibiotic susceptibility in hypermutators, and the hypermutators exhibit higher antibiotic resistance than normal bacteria (LeClerc et al., 1996; Oliver et al., 2002; Wilson et al., 2014). In a clinical isolate of *Acinetobacter baumannii*, D278N substitution in the core domain of MutS was found to confer a hypermutable phenotype and strong multiantibiotic resistance (Deihim et al., 2017). Similar to the findings in *A. baumannii*, all *Salmonella* hypermutators examined in our current study were resistant to multiple antibiotics (Wang et al., 2015a, b; Yang et al., 2010, 2013).

Many external factors can promote hypermutation in bacteria, including ultraviolet radiation, acid tolerance, and stress response, and the MMR system unquestionably acts as an intrinsic inhibitor to hypermutation (Ling et al., 2003; Woodford and Ellington, 2007; Hammerstrom et al., 2015; Dettman et al., 2016). However, for hypermutators, if the amino acid constitution of MutS, MutL, MutH, and UvrD is altered, the repair functions of the MMR system can be partially reduced or lost. As a consequence, deleterious mutations in the MMR system can lead to an increase in mutation opportunities for other genes, facilitating the survival of bacteria, especially in environments with stresses such as antibiotics (Hammerstrom et al., 2015; Mehta et al., 2019).

In the present study, four representative hypermutator strains with several mutations detected in MutS, MutL, and UvrD were further investigated. In additional to MutS, MutL, MutT, and UvrD mutations, here we report other genes, including *tol*C, *par*C, and *omp*F that were all mutated in the *Salmonella* hypermutators. We also found relatively high correlations between the extent of antibiotic resistance (i.e., the number of antibiotics resisted) and mutations in the main MMR genes (*mut*S, *mut*L, *mut*T, and *uvr*D) and antibiotic resistance genes (*par*C and *omp*F), although these correlations were not always significant.

Since these genes are related to antibiotic resistance, their mutation may be partially responsible for multidrug resistance in *Salmonella* hypermutators. Some connections might exist between *tol*C, *par*C, and *omp*F mutations and mutations in MutS, MutT, MutL, and UvrD. We found no significant correlation between mutation frequency and the number of mutations observed in most of the genes tested in this study, which is in agreement with other studies conducted in *Salmonella* hypermutators (Yang et al., 2009; Henderson-Begg et al., 2010; Wang et al., 2015a). Nonetheless, mutation frequency yielded the highest correlation coefficient with the extent of MutS mutation. No mutations were detected among most of the genes commonly associated with antibiotic resistance we evaluated, although a number of mutations were found in MutS, MutL, mutT, and uvrD. We therefore questioned whether mutation frequency and antibiotic susceptibility were influenced by the expression of genes in the MMR system, and those associated with antibiotic resistance in *Salmonella* hypermutators. To explore this, we employed RT-qPCR to quantify the relative expression levels of four MMR genes (*mut*S, *mut*L, *mut*H, and *uvr*D) and 15 functional genes associated with antibiotic resistance in *Salmonella* hypermutators.

In bacteria, MMR during DNA replication is initiated when MutS forms a homodimeric complex that can bind the mismatches; then, the MutS–mispair complex recruits a MutL homodimer to activate the MutH endonuclease (LeClerc et al., 1996; Modrich and Lahue, 1996; Hu et al., 2017). In the current study, although *mut*S, *mut*T, and *uvr*D were significantly upregulated, *mut*H was downregulated in the hypermutator strains 31 and 1171R. Further, clustering and correlation analysis revealed that *mut*H expression levels were negatively correlated with *mut*S, *mut*L, *mut*T, *acr*A, *acr*B, and *par*C expression levels (albeit not significant in some cases) and were also negatively correlated with the number of mutation sites and number of antibiotics resisted in each hypermutator. These results suggest the novel hypothesis that downregulation of *mut*H expression may be the primary reason leading to more mutation sites and increased multidrug resistance in *Salmonella* hypermutators. In addition, we found significant positive correlations between the number of mutation sites and expression of *mut*S, *mut*L, *gyr*A, and *mut*T. These relationships indicate that *mut*S and *mut*L mutations may hinder the assembly of a MutS–MutL–heteroduplex ternary complex (Modrich and Lahue, 1996; Hu et al., 2017; Li et al., 2018), resulting in decreased MutH expression in these hypermutator strains. However, mutation frequency did not correlate with expression levels of most genes tested in this study perhaps due to some unknown factors. Taken together, these results confirm that the number of mutation sites, mutation frequency, and increases in antibiotic resistance are interrelated in MMR-deficient *Salmonella* hypermutators.

According to the clustering and correlation analysis results, expression levels of the 19 functional genes in hypermutator strains 31 and 1171R were similar to levels in strains S8XC001a and 103D. Studies have indicated that inactivation of MarR increases expression levels of the antibiotic resistance gene *mar*A, as well as the transcription levels of efflux pump-encoding genes *acr*A, *acr*B, and *tol*C, thereby increasing ciprofloxacin resistance in bacteria (Alekshun and Levy, 1999; Vinué et al., 2016). In the current study, we observed low expression levels of *acr*A, *acr*B, and *tol*C in hypermutator strains 31, 1171R, and S8XC001a. However, it is possible that MMR deficiency and other mutations in those hypermutators may inactivate MarR or AcrR, thereby increasing the efflux activity of the multidrug efflux pump AcrAB-TolC (Alekshun and Levy, 1999; Yang et al., 2012). Moreover, the changes in expression of *omp*F and *omp*R may be responsible for the multidrug resistance phenotype of the hypermutators (Lindgren et al., 2003).

Bacteria can promptly respond to environmental changes by regulating genetic expression patterns through an elaborate response system and comprehensive gene expression network, which confer bacteria the ability to survive and adapt to new environments (Khil and Camerini-Otero, 2002; Li et al., 2018). In the case of *E. coli*, although DNA damage and mutagenesis occur due to oxidation, acid, cold, heat, or antibiotic usage, the bacteria effectively respond to stresses by differential protein synthesis (Eichelmann et al., 2006). In the current study, we observed an upregulation of common proteins (8/10) shared in the four *Salmonella* hypermutators. GO and KEGG enrichment analysis showed that RpsA (shared by strains 31 and S8XC001a) and RpsB (shared by strains 31 and 103D) were enriched in the translation and ribosome terms, whereas OmpA and GlpK (shared by strains 31 and 1171R) were enriched in the cellular response to DNA damage stimulus term.

Furthermore, interaction network analysis revealed that the ribosome pathway (involving RpsB, RpsA, RplL, RplI, RplO, and RpsF), which may be mediated by MMR deficiency, was a critical factor for the high mutation frequency and multidrug resistance in the *Salmonella* hypermutators. Based on the 2-DE and MS results, five of the six ribosome-related proteins (except for RplL) were significantly upregulated in the hypermutators. The bacterial ribosome is comprised of three ribosomal RNAs (rRNAs: 16S, 23S, and 5S) and approximately 54 ribosomal proteins, which are commonly targets of antibiotics; if antibiotics are used, the synthesis of bacterial ribosomal proteins can be blocked, thus mitigating the antibacterial effect (Poehlsgaard and Douthwaite, 2005; Voorhees and Ramakrishnan, 2013). Here, the upregulated expression of ribosomal proteins indicated that the MMR-deficient *Salmonella* hypermutators might possess powerful protein synthesis response and could exhibit rapid compensatory responses against strong antibiotic stimulation and adverse environmental changes.

Gene Ontology enrichment analysis revealed that seven upregulated proteins (GlpK, OmpA, YciE, YciF, NagE, DeoA, and PurA) and the MutS-protein-encoding gene were associated with cellular response to DNA damage stimulus. Among the *Salmonella* hypermutators, overexpression of those proteins might contribute to multidrug resistance and high mutation frequency, promoting survival in the presence of antibiotic(s). Interestingly, multiple proteins involved in stress response, including oxidation (Lpd, Icd, AhpC, SodA, and Dps) (Seaver and Imlay, 2001; Gundlach and Winter, 2014; Krisko et al., 2014), heat (DnaK and SodA) (Arsène et al., 2000), and chemical stimulus (SodA, AhpC, and DnaK) (Glebes et al., 2015), were detected by MS in the four hypermutators. These proteins can coordinate gene expression networks in bacteria in response to external stresses including antibiotic exposure, stressful conditions, changed environment, and extreme nutrient limitation, thereby enabling the cells to adapt to adverse environmental conditions (Taddei et al., 1997; Woodford and Ellington, 2007).

In summary, mutation frequency and antibiotic resistance in *Salmonella* hypermutators are directly or indirectly associated with the mutation and expression levels of *mut*S, *mut*T, *mut*L, and *uvr*D, genes involved in the MMR system. Proteins associated with cellular response to DNA damage stimulus and stress response were detected as being differentially or specifically expressed in the hypermutators. The ribosome pathway appeared to be the critical factor for high mutation frequency and multidrug resistance mediated by MMR deficiency in *Salmonella* hypermutators. Multiple pathways likely coexist in the *Salmonella* hypermutators, jointly influencing their mutation frequency and antibiotic resistance.

## Data Availability Statement

All datasets generated for this study are included in the article/Supplementary Material.

## Author Contributions

HS contributed to experiment design, doing experiment, and writing the manuscript. JH contributed to data analysis. ZH, ML, ZL, and QZ contributed to sample collection and analysis. JZ and JY contributed to data analysis. SC contributed to manuscript revising. BY contributed to experiment design and manuscript revising.

## Conflict of Interest

The authors declare that the research was conducted in the absence of any commercial or financial relationships that could be construed as a potential conflict of interest.

## Footnotes

1 https://www.ncbi.nlm.nih.gov/Structure/cdd/wrpsb.cgi

2 http://www.ncbi.nlm.nih.gov/BLAST/

3 https://www.uniprot.org/

4 https://biocyc.org/

5 https://david.ncifcrf.gov/summary.jsp

6 http://www.genome.jp/kaas-bin/kaas_main

7 http://string-db.org/

8 https://cytoscape.org/

9 http://cran.r-project.org/
