# Supplementary figures and images for: Genes and Proteomes Associated With Increased Mutation Frequency and Multidrug Resistance of Naturally Occurring Mismatch Repair-Deficient Salmonella Hypermutators
Source: Front Microbiol. 2020 May 8;11:770. doi: 10.3389/fmicb.2020.00770 (PMC7225559; doi:10.3389/fmicb.2020.00770)

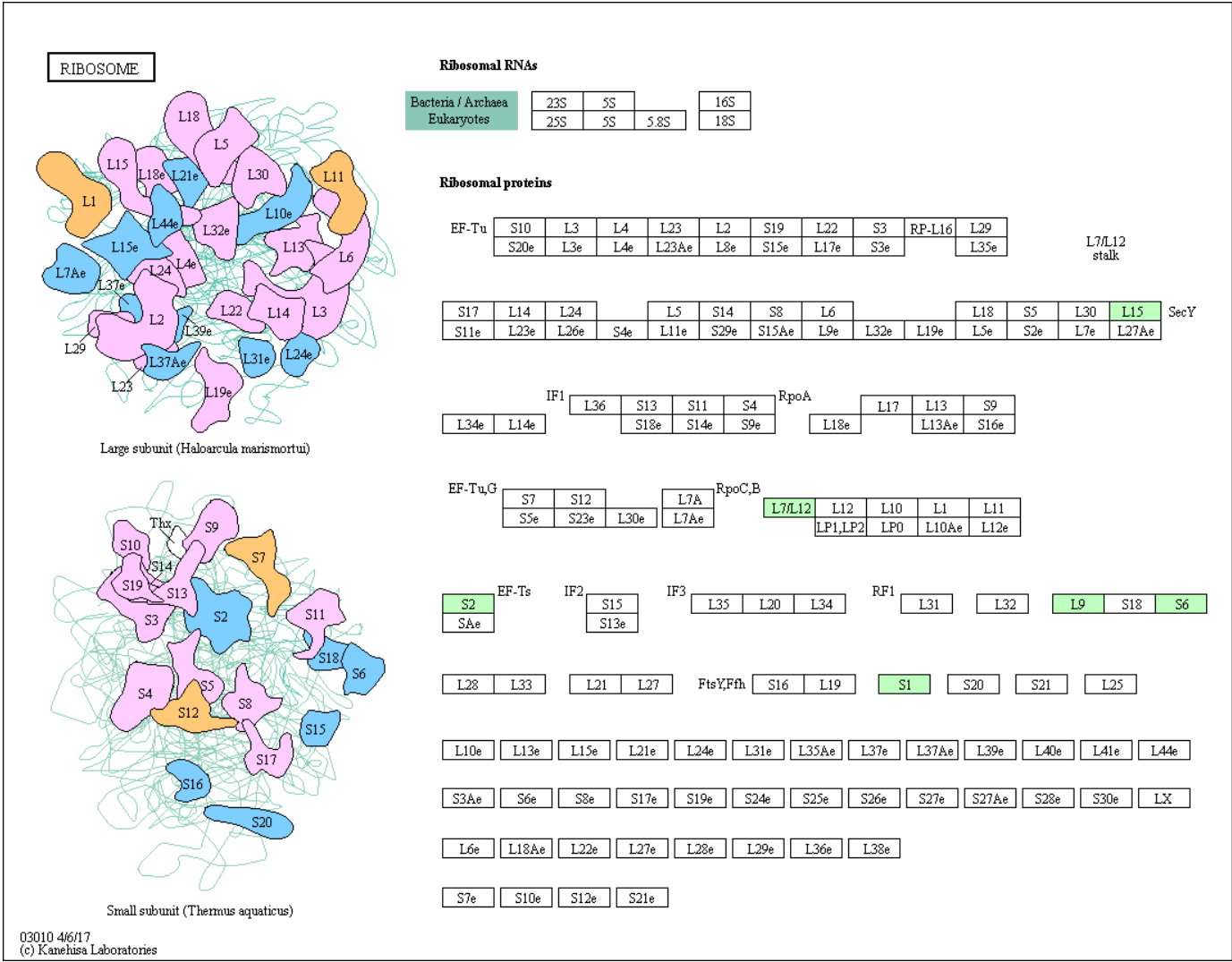

Supplement: Supplementary file 1 [file Data_Sheet_1.PDF]
