# Supplementary material for: Genes and Proteomes Associated With Increased Mutation Frequency and Multidrug Resistance of Naturally Occurring Mismatch Repair-Deficient Salmonella Hypermutators
Source: Front Microbiol. 2020 May 8;11:770. doi: 10.3389/fmicb.2020.00770 (PMC7225559; doi:10.3389/fmicb.2020.00770)
Supplement: Supplementary file 2 [file Table_1.DOCX]

**Supplemental Table S1 | Primers used in PCR amplification and Primers used in RT-PCR**

| Genes | Forward Primer (5’→3’) | Reverse Primer (5’→3’) | Size (bp) |
| --- | --- | --- | --- |
| Primers for PCR amplification | | |  |
| *gyr*A | CGTTGGTGACGTAATCGGTA | CCGTACCGTCATAGTTATCC | 251 |
| *gyr*B | GCGCTGTCCGAACTGTACCT | TGATCAGCGTCGCCACTTCC | 181 |
| *acr*A | CGGTCGTTCTGATGCTCTCA | GCCCTGTTGTGGAACCAGTA | 885 |
| *acr*B | AAGAGCACGCATCACTACAC | CGCTTCGGACATCACGTAAA | 768 |
| *omp*F | ATGAAGCGCAAAATCCTGGCAGCG | TCAGAACTGGTAAGTAAATACCGAC | 1089 |
| *par*C | CTATGCGATGTCAGAGCTGG | TAACAGCAGCTCGGCGTATT | 270 |
| *par*E | TCTCTTCCGATGAAGTGCTG | ATACGGTATAGCGGCGGTAG | 240 |
| *tol*C | CAGACGCTGATCCTCAATAC | TGCTGATGGAGGCGTTAATA | 717 |
| *mar*A | CGCAACACTGACGCTATTAC | TTCAGCGGCAGCATATAC | 358 |
| *mar*R | ATTCCGCTGGGTCGCTTGA | AGCGCCGCGGTGTTACTC | 1285 |
| *acr*E | TTTTCACTCCTGCCCTCA | AGTTGTTGCACGGTAGCC | 605 |
| *mut*L | GATTATCAGTCTGGGATTTCGC | TCGCCTTGCTTACATCATTC | 1608 |
| *mut*H | GATAAACTCTGCCCATACGG | AATAGCGGCGACAATACGCT | 840 |
| *mut*T | ATCGCGGTTGGGATTATCCG | TAAAGCGCAAATTGGCGTAA | 400 |
| *uvr*D | CTTACCTGCTCGACAGCCTT | GGCACTATGAGAGTCGGTCA | 2100 |
| *omp*R | AGCGGCATCGGTTCATCT | GAGCAGGGCTTCCAGGTT | 407 |
| *sox*S | TTCATCGCCTGGCTACAA | ACTCGCCCGCAGACAAAA | 431 |
| *hil*A | CTGTCGGAAGATAAAGAGC | CAGAAATGGGCGAAAGTA | 859 |
| *dna*Q | GGTGCGGTTGAGGTGATA | TCGCCCGAAGGATGATTT | 673 |
| Primers for RT-PCR | |  |  |
| *gyr*A | ACGTATTGGGCAATGACTGG | GGAGTCGCCGTCAATAGAAC | 190 |
| *gyr*B^a^ | CAAACTGGCGGACTGTCAGG | AGCCCAGCGCGGTGATCAGC | 211 |
| *acr*A | CCCCAACTTCTGGCATCT | ATTTGAAATCGGACACTCG | 153 |
| *acr*B | ATCATCGCCATCGCTTCA | ATTCTCCGCATTCTCCTCTT | 132 |
| *acr*E | AACTTCACCGAAGGTAGCG | GCCGACCAGTGGAACATAG | 165 |
| *par*C | CGTCTATGCGATGTCAGAGC | TAACAGCAGCTCGGCGTATT | 264 |
| *par*E | GTCAATGTGCGGCATTTGTT | ATCCCCTTCCACAAGGAACA | 241 |
| *tol*C | CAGCAATAACGGCAATCC | CCAGACCTACAAGGGCACA | 83 |
| *mar*A | ATCCGCAGCCGTAAAATGAC | TGGTTCAGCGGCAGCATATA | 199 |
| *mar*R | AGGATTCGGCAGTCTTTC | GTGCTTTGCTCGATACGC | 138 |
| *mut*S | CTATCTGGCAGGATGGTAAAG | AATCTCAAACTCCCACAACG | 215 |
| *mut*L | GCCTTTACCCTTACGACAACA | GACATAGCCGCTCAACATCC | 188 |
| *mut*H | CGTCCTCTGGAAACAACCT | CTGCCTGTCCTCTTCTTCACTC | 198 |
| *mut*T | GGCGAAACACCAGAGCAG | AGCGTTCAACCAGCCAAA | 142 |
| *uvr*D | ACGCTAACCTATGCCGAAAC | TCTTCCACGCACTCCTCC | 98 |
| *omp*F | GCCAGACGGACCAGATTC | TGCCGACCAGACTTATGC | 143 |
| *omp*R | CTTCGCCGTGACCATAAT | GCTGACCCGTGAATCTTT | 127 |

^a^ *gyr*B was reference gene
