# Supplementary material for: Genes and Proteomes Associated With Increased Mutation Frequency and Multidrug Resistance of Naturally Occurring Mismatch Repair-Deficient Salmonella Hypermutators
Source: Front Microbiol. 2020 May 8;11:770. doi: 10.3389/fmicb.2020.00770 (PMC7225559; doi:10.3389/fmicb.2020.00770)
Supplement: Supplementary file 3 [file Table_2.DOCX]

**Supplemental Table S2 | Amino acid substitution and of 19 functional genes associated with antibiotic resistance and DNA replication repair in four *Salmonella* hypermutators**

| **Protein** | **Function** | **31** | | **1171R** | | **S8XC001a** | | **103D** | |
| --- | --- | --- | --- | --- | --- | --- | --- | --- | --- |
|  |  | **Amico acid substitution** | **Relative expression level** | **Amico acid substitution** | **Relative expression level** | **Amico acid substitution** | **Relative expression level** | **Amico acid substitution** | **Relative expression level** |
| AcrA | Efflux pump | ND | -0.28±0.04 | ND | -0.41±0.13 | ND^a^ | 0.3±0.39 | ND | 0.66±0.18 |
| AcrB | Efflux pump | ND | -0.66±0.02 | ND | -0.73±0.08 | ND | -0.47±0.61 | ND | 0.23±0.03 |
| AcrE | Similar to *Escherichia coli* transmembrane protein affects septum formation and cell membrane permeability | ND | 0.82±0.23 | ND | -0.22±0.35 | ND | -0.59±0.58 | ND | 0.76±0.3 |
| TolC | Outer membrane protein | ND | -0.42±0.03 | Val231Ile | -0.37±0.02 | ND | 1.17±0.37 | Ser290Ala | 0.32±0.09 |
|  |  |  |  |  |  |  |  | Thr291Ala |  |
| MarA | Multiple antibiotic resistance | ND | 0.3±0.06 | ND | 0.28±0.08 | ND | -0.04±0.04 | ND | 0.71±0.1 |
| MarR | Multiple antibiotic resistance | ND | 0.6±0.42 | ND | 1.08±0.25 | ND | 0.19±0.28 | ND | 3.97±0.09 |
| DnaQ | Encodes the proofreading 3' exonuclease of DNA polymerase III holoenzyme | ND | 3.76±0.08 | ND | 0.23±0.03 | ND | -0.21±0.11 | ND | 0.47±0.02 |
| GyrA | Gyrase subunit | ND | 0.37±0.23 | ND | 0.62±0.08 | ND | -0.15±0.16 | ND | -0.06±0.28 |
| ParC | Topoisomerase IV subunit | Ser80Arg | 1.69±0.09 | Ser80Arg | 1.22±0.49 | Glu130Gly | 0.74±0.25 | ND | 0.6±0.16 |
|  |  |  |  | Glu130Ser |  |  |  |  |  |
| ParE | Topoisomerase IV subunit | ND | 0.59±0.17 | ND | -0.42±0.06 | ND | -0.22±0.03 | ND | 0.39±0.01 |
| MutS | DNA mismatch recognition | Val246Ala | 1.22±0.39 | Val246Ala | 1.08±0.62 | Val421Phe | 0.3±0.21 | Val246Ala | 0.33±0.88 |
| MutL | Stimulates MutS, MutH and Vsr activity | Ala235Thr | 0.37±0.43 | Ala235Thr | 0.64±0.48 | Gln249Pro | -0.01±0.18 | Gln249Pro | 0.09±0.57 |
|  |  | Gln249Pro |  | Gln249Pro |  |  |  |  |  |
|  |  | Gly292Val |  | Gly292Val |  |  |  |  |  |
|  |  | Gln304Leu |  | Gln304Leu |  |  |  |  |  |
|  |  | Thr311Ala |  | Thr311Ala |  |  |  |  |  |
|  |  | Ala318Thr |  | Ala318Thr |  |  |  |  |  |
|  |  | Ser324Pro |  | Ser324Pro |  |  |  |  |  |
|  |  | Thr326Ala |  | Thr326Ala |  |  |  |  |  |
|  |  | Lys403Gln |  | Lys403Gln |  |  |  |  |  |
| MutH | Endonuclease | ND | -0.91±0.01 | ND | -0.96±0.03 | ND | -0.21±0.24 | ND | -0.2±0.52 |
| MutT | Nucleoside triphosphatase | Ser111Asn | 2.33±0.15 | Ser111Asn | 1.86±0.21 | ND | 0.11±0.12 | Glu91Asp | 1.22±0.75 |
| UvrD | DNA helicase II | Gln 50Lys | 1.45±0.59 | Val440Ala | -0.07±0.49 | ND | -0.66±0.3 | ND | 0.58±0.84 |
|  |  | Gln260His |  |  |  |  |  |  |  |
|  |  | Val440Ala |  |  |  |  |  |  |  |
| OmpF | The outer membrane porin F gene | Gly307Asp | 0.41±0.23 | 271 deletion of Asn | 0.62±0.38 | ND | -0.49±0.08 | Gly307Asp | 0.58±0.38 |
|  |  |  |  | Gly307Asp |  |  |  |  |  |
| OmpR | Regulates transcription of outer membrane porin genes *omp*C/F | ND | -0.09±0.06 | ND | 0.01±0.16 | ND | -0.32±0.09 | ND | 0.27±0.19 |
| HilA | Activates the expression of invasion genes and activates the expression of prgHIJK which is part of the pathogenicity islandⅠtype III secretion system | Gln118Leu | 0.59±0.05 | Gln118Leu | 8.09±1.42 | ND | -0.48±0.13 | ND | 5.66±0.03 |
| SoxS | Regulates genes involved in response to oxidative stress | ND | -0.06±0.19 | ND | 5.66±1.1 | ND | -0.26±0.03 | ND | 0.59±0.07 |
| No. of mutation sites | | 17 | | 18 | | 3 | | 6 | |
| No. of antibiotic resisted | | 10 | | 11 | | 10 | | 4 | |
| Mutation frequency | | 1.08×10^-3^ | | 3.39×10^-4^ | | 5.46×10^-2^ | | 1.32×10^-3^ | |

^a^ ND indicated amino acid substitution was not detected.
