# Supplementary material for: Genes and Proteomes Associated With Increased Mutation Frequency and Multidrug Resistance of Naturally Occurring Mismatch Repair-Deficient Salmonella Hypermutators
Source: Front Microbiol. 2020 May 8;11:770. doi: 10.3389/fmicb.2020.00770 (PMC7225559; doi:10.3389/fmicb.2020.00770)
Supplement: Supplementary file 6 [file Table_5.DOCX]

**Supplemental Table S5 | Mass Spectrometry identification of 68 protein species from four *Salmonella* hypermutators**

| Isolate | Spot number | Expression | Quantitation | Quantitation  (*Salmonella* Typhimurium LT2) | Protein name | Abbreviation |
| --- | --- | --- | --- | --- | --- | --- |
| 31 | 5501 | Specific | 6547.1 |  | Peptidase PmbA | PmbA |
|  | 5303 | Up-regulated | 12414.4 | 4843.8 | Glycerophosphoryl diester phosphodiesterase periplasmic precursor | GlpQ |
|  | 5203 | Up-regulated | 4584.3 | 1934.9 | Adenylate kinase | Adk |
|  | 5103 | Up-regulated | 2632.7 | 1053.3 | Periplasmic protein | YraP |
|  | 4201 | Up-regulated | 2608.2 | 1198.9 | Ribose-phosphate pyrophosphokinase | Prs |
|  | 4102 | Up-regulated | 3690.8 | 1684.3 | 30S ribosomal protein S6 | RpsF |
|  | 4001 | Up-regulated | 10170.2 | 3551.7 | Global DNA-binding transcriptional dual regulator H-NS | Hns |
|  | 3401 | Up-regulated | 7453.9 | 2165.5 | DNA-directed RNA polymerase subunit alpha | RpoA |
|  | 1804 | Up-regulated | 9306 | 2517.8 | Molecular chaperone DnaK | DnaK |
|  | 1102 | Up-regulated | 2195.9 | 731.9 | 30S ribosomal protein S1 | RpsA |
|  | 1101 | Up-regulated | 7271.3 | 1822.5 | SSU ribosomal protein S2p (SAe) | RpsB |
|  | 0103 | Up-regulated | 3692.2 | 541.8 | General stress protein 16U | TerD |
|  | 4601 | Specific | 10230.6 |  | Glycerol kinase | GlpK |
|  | 3301 | Specific | 3005.2 |  | OmpA, partial | OmpA |
|  | 2104 | Specific | 1778 |  | Outer membrane protein X | OmpX |
|  | 2102 | Specific | 2979 |  | Transcriptional regulator | DksA |
|  | 5401 | Down-regulated | 12143.2 | 29206.1 | Elongation factor Tu, partial | TufA |
|  | 3502 | Down-regulated | 5277.9 | 12620 | Clp protease ClpX | ClpX |
|  | 3003 | Down-regulated | 2478.3 | 5028.8 | Autonomous glycyl radical cofactor GrcA | GrcA |
|  | 1507 | Down-regulated | 1051.5 | 8538.8 | F0F1 ATP synthase subunit beta | AtpD |
|  | 1502 | Down-regulated | 7546.1 | 16927 | Flagellin | FljB |
| 1171R | 9103 | Up-regulated | 1691.4 | 95.7 | Superoxide dismutase | SodA |
|  | 9003 | Up-regulated | 1818.8 | 421.8 | 50S ribosomal protein L9 | RplI |
|  | 9001 | Up-regulated | 5569.8 | 1699.5 | Putative sigma(54) modulation protein | Hpf |
|  | 8704 | Up-regulated | 5459.4 | 1810.6 | Dihydrolipoamide dehydrogenase | LpdA |
|  | 8203 | Up-regulated | 3951.3 | 1667.1 | Succinyl-CoA synthetase subunit alpha | SucD |
|  | 6701 | Up-regulated | 13802 | 4120.1 | Glycerol kinase | GlpK |
|  | 5502 | Up-regulated | 29853.8 | 11026.7 | Elongation factor Ts | Tsf |
|  | 5302 | Up-regulated | 2656.8 | 901.4 | NAD+ synthetase | NadE |
|  | 4201 | Up-regulated | 10952.4 | 5310.2 | Short-chain dehydrogenase | UcpA |
|  | 4102 | Up-regulated | 4676.5 | 1618.8 | DNA starvation/stationary phase protection protein Dps | Dps |
|  | 1806 | Up-regulated | 6497.3 | 2802.8 | Molecular chaperone DnaK | DnaK |
|  | 0101 | Up-regulated | 3272.1 | 1008.7 | PTS glucose transporter subunit IIA | NagE |
|  | 9009 | Specific | 782.4 |  | 50S ribosomal protein L15 | RplO |
|  | 8204 | Specific | 5713.2 |  | Phosphoglyceromutase | GpmA |
|  | 4301 | Specific | 3245.8 |  | OmpA, partial | OmpA |
|  | 4107 | Specific | 3044.8 |  | Periplasmic protein | YraP |
|  | 0602 | Specific | 2841.7 |  | Flagellin | FljB |
| S8XC001a | 4602 | Specific | 12978.3 |  | Endo-1,4-D-glucanase | Tsf |
|  | 4503 | Up-regulated | 17309.1 | 6894.3 | Adenylosuccinate synthetase | PurA |
|  | 3402 | Up-regulated | 21853.8 | 7455.2 | Phosphoglycerate kinase | Pgk |
|  | 3201 | Up-regulated | 16423.3 | 7825.2 | Short-chain dehydrogenase | UcpA |
|  | 2101 | Up-regulated | 9299.2 | 4275.9 | Periplasmic protein | YraP |
|  | 1802 | Up-regulated | 9812.5 | 3567.1 | Molecular chaperone DnaK | DnaK |
|  | 1701 | Up-regulated | 10613.2 | 3818.9 | 30S ribosomal protein S1 | RpsA |
|  | 0002 | Up-regulated | 4509.5 | 2214.6 | Mn-containing catalase | STM1731 |
|  | 4106 | Specific | 3855.7 |  | Protein yciF | YciF |
|  | 3504 | Specific | 8198.3 |  | Isocitrate dehydrogenase | IcdA |
|  | 0303 | Specific | 2439.7 |  | Flagellar synthesis: phase 2 flagellin (filament structural protein) | FljB |
| 103D | 9005 | Specific | 1801.6 |  | Cold shock protein CspE | CspE |
|  | 4101 | Up-regulated | 4685.5 | 2056.9 | YciE | YciE |
|  | 0002 | Up-regulated | 744.9 | 1996.1 | Alkyl hydroperoxide reductase subunit C | AhpC |
|  | 2402 | Up-regulated | 5675.2 | 1118.6 | Thymidine phosphorylase | DeoA |
|  | 1102 | Up-regulated | 3520.4 | 1683.1 | SSU ribosomal protein S2p (SAe) | RpsB |
|  | 0501 | Specific | 3837.2 |  | Transcription termination factor Rho | Rho |
|  | 5702 | Down-regulated | 746.7 | 1749.9 | Lysyl-tRNA synthetase | LysS |
|  | 5303 | Down-regulated | 5532.5 | 19143 | Glycerophosphodiester phosphodiesterase | GlpQ |
|  | 1502 | Down-regulated | 1543 | 14372.5 | Alpha-helical coiled coil protein, partial |  |
|  | 0002 | Down-regulated | 744.9 | 1996.1 | 50S ribosomal protein L7/L12 | RplL |
